# Evaluation on physiological biomarkers pre and during gestational period in immunosuppressed Wistar rats with mycophenolate sodium

**DOI:** 10.1186/1758-5996-7-S1-A69

**Published:** 2015-11-11

**Authors:** Amanda Lima Deluque, Nagilla Orleanne Lima do Carmo, Elisangela Miranda de Jesus Lisboa, Gilsielle Benício Jaco, Betina Beatriz Mielke, Madileine Francely Americo, Luciana Aparecida Cora, Maria do Carmo Borges Teixeira, Kleber Eduardo de Campos

**Affiliations:** 1 Universidade Federal de Mato Grosso, Barra do Garças, Brazil

## Background

The use of medication during pregnancy should be taken with caution, especially because of its implications for maternal and fetal health. The mycophenolate sodium (MFS) is used to avoid possible rejection of transplanted tissues and organs by inhibiting T and B lymphocyte proliferation.

## Objective

To evaluate the effects of maternal immunosuppression by MFS on physiological parameters in rats.

## Materials and methods

The rats were divided into three groups: rats treated with water (CONT=10) rats orally treated with MFS (20mg/Kg) daily for 15 days until a positive diagnosis of pregnancy (MICO-1=10) and rats orally treated with MFS (20mg/Kg) daily for 15 days prior to and during 21 days of pregnancy (MICO-2=10). It was weekly evaluated body weight, blood glucose, and water and food intake (before and during pregnancy). Statistical significance was p <0.05.

## Results

In the pre-pregnancy period, although there were no changes in body weight values, the continuous treatment with MFS decreased food and water consumption and also increased blood glucose (MICO-2). During pregnancy period, these rats presented a reduction of the body weight, maintaining a mild hyperglycemia level (about 170 mg/dL)

## Conclusion

The continued use of MFS during pregnancy can be considered toxic, damaging the maternal health, lead to mild hyperglycemia state plus some toxic data (low food and water intake). This study shows that MFS use is not recommended during pregnancy freely and should be replaced by another immunosuppressant.

